# Approach and Management of Pregnancies with Risk Identified by Non-Invasive Prenatal Testing

**DOI:** 10.3390/jpm14040366

**Published:** 2024-03-29

**Authors:** Miruna Gug, Adrian Rațiu, Nicoleta Andreescu, Simona Farcaș, Sorina Laitin, Cristina Gug

**Affiliations:** 1 Doctoral School, “Victor Babeş” University of Medicine and Pharmacy, 300041 Timisoara, Romania; miruna.gug@umft.ro; 2 Medical Genetics Office Dr. Gug, 300200 Timisoara, Romania; cristina.gug@umft.ro; 3 Department of Obstetrics and Gynecology II, “Victor Babes” University of Medicine and Pharmacy, 300041 Timisoara, Romania; ratiu.adrian@umft.ro; 4 Timisoara Municipal Emergency Clinical Hospital, 300202 Timisoara, Romania; 5 Department of Microscopic Morphology, Discipline of Genetics, “Victor Babeş” University of Medicine and Pharmacy, 300041 Timisoara, Romania; andreescu.nicoleta@umft.ro; 6 Genomic Medicine Centre, “Victor Babeș” University of Medicine and Pharmacy, 300041 Timisoara, Romania; 7 Department of Infectious Diseases, Discipline of Epidemiology, “Victor Babeş” University of Medicine and Pharmacy, 300041 Timisoara, Romania; laitin.sorina@umft.ro

**Keywords:** non-invasive prenatal testing, cffDNA, prenatal diagnostics, fetal aneuploidy, CNVs

## Abstract

This study represents our second investigation into NIPT, involving a more extensive patient cohort with a specific emphasis on the high-risk group. The high-risk group was subsequently divided into two further groups to compare confirmed cases versus unconfirmed via direct methods. The methodology encompassed the analysis of 1400 consecutive cases from a single genetic center in western Romania, where NIPT was used to assess the risk of specific fetal chromosomal abnormalities. All high-risk cases underwent validation through direct analysis of fetal cells obtained via invasive methods, including chorionic villus sampling and amniocentesis. The confirmation process utilized QF-PCR, karyotyping, and SNP-Array methods customized to each case. Results: A high risk of aneuploidy at NIPT was identified in 36 out of 1400 (2.57%) cases and confirmed in 28 cases. The study also detected an increased risk for copy number variations (CNVs) in 1% of cases, confirmed in two instances involving one large microdeletion and one large microduplication. Trisomy 21 was the exclusive anomaly where NIPT confirmed all cases with identified risk. High-risk NIPT results which were not validated by invasive methods, were classified as false positives; parents in these cases determined to continue the pregnancy. In conclusion, NIPT can serve as a screening method for all pregnancies; however, in high-risk cases, an invasive confirmation test is strongly recommended.

## 1. Introduction

In 1997, the detection of cell-free fetal DNA (cffDNA) in maternal plasma was announced for the first time. Non-invasive prenatal testing (NIPT) was initially launched in Hong Kong [1] shortly after it was commercially introduced in the USA [2]. It subsequently spread to many countries through various companies offering different types of NIPT tests to millions of pregnant women.

In 2014, the first cases in Romania underwent testing, and the preliminary findings were previously published [3]. Initially, NIPT was used to detect common fetal aneuploidies [4] and later expanded to include screening for rare fetal aneuploidies, incorporating a growing repertoire of 92 chromosomal number variations (CNVs). The implementation of the expanded NIPT panel should be accompanied by pre- and post-test genetic counseling by experts [5].

Next-generation sequencing (NGS) technologies have significantly increased the method’s accuracy of detecting fetal aneuploidies to nearly 90–99% [4], surpassing maternal biochemical screening and fetal sonography, which have a detection rate of 50–95% [6]. Sex chromosome aneuploidies identified by NIPT have a lower detection rate, the accuracy being less than 50% for monosomy X. [7]. The precision in detecting rare autosomal aneuploidies and notable copy number variations (CNVs) remains relatively limited.

In 2012, NIPT was recommended by the American College of Obstetricians and Gynecologists only for women previously classified as high risk by traditional screening [8]. However, with an increasing number of NIPTs performed worldwide, after 4 years expert opinion has evolved. The current international guidelines now recommend the use of NIPT for prenatal screening during pregnancy for all women, regardless of the pre-determined risk of occurrence of fetal anomalies [9]. In the last decade, NIPT has become one of the most fascinating research fields in molecular medicine, with a rapid implementation in medical practice.

## 2. Materials and Methods

A retrospective study was undertaken utilizing data obtained from a single private genetic center, which collects cases from the western region of Romania. Between September 2014 and December 2023, NIPT was utilized for 1400 Caucasian women. We note that 13 twin pregnancies and 85 pregnancies resulting from in vitro fertilization (IVF) were included in this study. For each pregnancy, an obstetrician performed ultrasonography assessments to determine the number of fetuses and gestational age, followed by blood sampling for NIPS.

**Figure 1:**
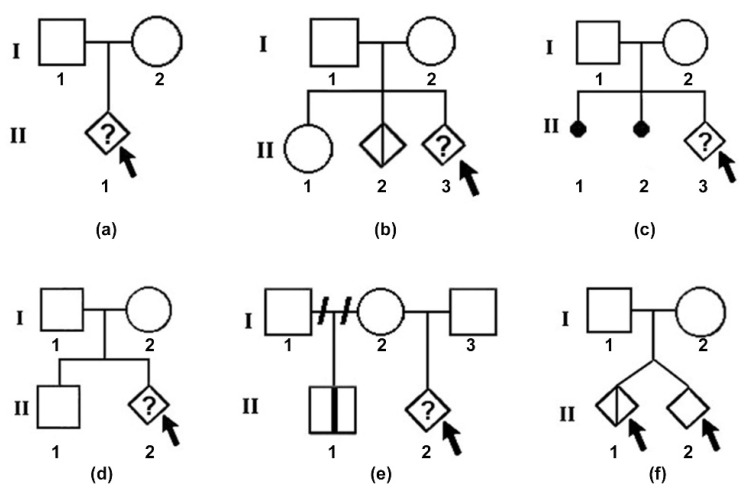
Example of pedigrees performed during genetic counselling prior to NIPT (the arrow indicates the pregnancy in disscution): (**a**) a couple at first pregnancy (gesta 1, para 0), pregnant with age > 35 years; (**b**) a couple with a healthy child, a pregnancy stopped for medical reasons for a fetus with trisomy 18, and currently pregnant at pregnancy nr 3 (gesta 3, para 1)—will do NIPT test; (**c**) a couple who had 2 previous pregnancies stopped for unknown reasons and currently at pregnancy nr 3 (gesta 3, para 0)—will do NIPT test; (**d**) a couple with a healthy child and currently at pregnancy nr 2(gesta 2, para 0—will do NIPT; (**e**) a pregnant woman (gesta 2, para 1) in her second marriage and because she has a child with trisomy 21 will do NIPT at current pregnancy; (**f**) a couple with a twin pregnancy via in vitro fertilization and did NIPT, with risk of trisomy 21 confirmed in one fetus after double amniocentesis followed by QF-PCR analysis.

A geneticist provided counseling to the couples on two to three occasions. In pre-test counseling, maternal age (MA), gestational age (GA), obstetric history, biochemical and ultrasound markers results, and hereditary diseases in the family were noted. The geneticist also constructed family pedigrees during this first session. Following the reception of the NIPT results, a second genetic counseling session was held to inform the parents of the implications of the identified risk. In the high-risk cases, we recommended and performed a genetic diagnosis through an invasive method, either chorionic villus sampling (CVS) or amniocentesis, followed by a combination of tests: quantitative fluorescent polymerase chain reaction (QF-PCR), microarray, and karyotyping. Subsequently, the patients benefited from a third genetic counseling session to better understand the significance of the results (Figure 1).

NIPT requires the extraction of cffDNA from a sample of maternal blood that is drawn into specific tubes: ccffDNA BCT™ tubes (Streck). NGS technology is used and low-coverage whole-genome sequencing is performed. The Nifty test (BGI Laboratory) allows the identification of six autosomal aneuploidies (chromosomes 13, 18, 21, 9, 16, and 22), sex chromosome aneuploidies, and 92 microdeletion-duplication syndromes. Between 2014 and 2023, 644 pregnant women were tested with the Nifty-pro test. The NIPS test, offered by the Invitae Laboratory USA, allows the detection of three common aneuploidies and six microdeletion syndromes. It was used in 756 cases from 2019 to 2022. Both tests can ascertain the presence of the Y chromosome and necessitate a minimum gestational age of 10 weeks. In twin pregnancies, only the Y chromosome and the trisomy 21, 18, and 13 results are valid. 

All cytogenetic techniques were performed in our own laboratory. In the case of obtaining fetal cells through invasive methods, cell cultures were initiated in two flasks with AmnioMax TM-II complete medium containing fetal bovine serum, gentamicin, and L-glutamine (Gibco-Thermo Fisher, Waltham, MA, USA) and placed in an incubator (37 °C, 5% CO_2_). The cell culture was monitored daily and cell growth, alongside number of mitoses, were noted. The cell culture media was changed on the fifth day. Long-term cultures were performed (12 days), and routine techniques harvested the cells. GTG banding (resolution 400–550-bands/haploid set) was used and at least 20 metaphases per sample were analyzed. Results were regularly available within 14–18 days. The cytogenetic results were formulated according to the International System for Human Cytogenetic Nomenclature (ISCN 2016) [10].

In cases classified at high risk of aneuploidy for chromosomes 13, 18, 21, X, and Y, the samples were analyzed using the rapid QF-PCR test with the Devyser Compact v3 kit that has been validated for DNA extraction from amniotic fluid and from chorionic villus sampling (CVS). The ABI GeneAmp^®^ System 9700 was used for amplification. Detection was performed on Applied Biosystems Genetic Analyzers (ABI 3500, Thermo Fisher Scientific Inc. (NYSE: TMO) Waltham, MA, USA). QF-PCR tests and microarray analyses were performed by a partner laboratory (CytoGenomic Medical Laboratory, Bucharest, Romania). The Affymetrix CytoScan 750 K microarray platform was used, which allows the detection of genetic imbalances, losses (deletions/microdeletions), or gains (duplications/microduplications) known as CNVs (copy number variants) larger than 100 kb, genome wide. The SNPs micro-array method was performed on samples extracted from amniotic fluid or chorionic villi.

The statistical analysis was organized based on the comparison between the NIPT-identified risk group and the group where the risk was verified. Positive predictive value (PPV) and negative predictive value (NPV) indicators were used and were calculated for both aneuploidies and microdeletions, considering confirmed cases, false positives, and false negatives. The probability that a positive result is correct is PPV, and the probability that a negative result is correct represents the NPV. For the numerical variables, the descriptive statistics were calculated, and histograms and column graphs were made. Public databases (DECIPHER, OMIM, NCBI) were utilized for data interpretation. Statistical analysis was performed as described earlier [3].

## 3. Results

### 3.1. Making a Family Pedigree

Creating a family pedigree played a crucial role in genetic counseling, aiding the geneticists in elucidating the situation and identifying pre-existing risks. Figure 1 showcases several illustrative cases for reference.

### 3.2. Distribution of Cases by Years

**Figure 2:**
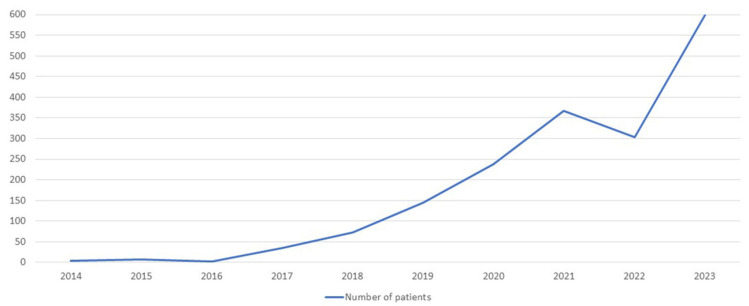
Distribution of cases in the studied period (2014–2023).

A total of 1400 pregnant women underwent one of the two NIPT tests (NIPS or Nifty-pro) at a private genetic center in Timișoara, Romania, between September 2014 and December 2023. In Romania, there is an increasing interest in this type of testing, highlighted by the large number of investigations carried out in pregnant women (Figure 2). 

### 3.3. Confirmed Versus High-Risk Cases at NIPT

**Figure 3:**
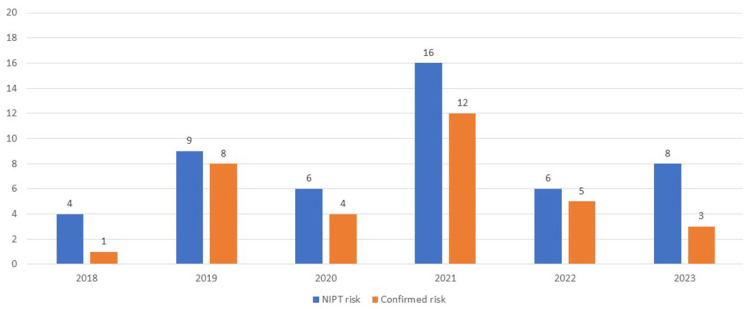
Distribution of cases identified as at risk during NIPT screening compared to cases confirmed as abnormal by direct diagnostic methods.

In the context of NIPT, 47 cases displaying a high risk were detected, with 3 of them having combined anomalies. From 2018 to 2023, numerous invasive procedures (such as CVS or amniocentesis) were performed with the aim of having a direct diagnostic method. The confirmed cases for each year are depicted in Figure 3.

### 3.4. Twin Pregnancies

Thirteen cases with twin pregnancies were registered, for which it was only possible to test for common trisomies and the Y chromosome. A heightened risk for fetal aneuploidy was identified in two cases, leading to amniocentesis. In the first case, fetal karyotyping revealed a male fetus with trisomy 21 and a normal female fetus. The second case, assessed through the QF-PCR method, indicated a female fetus with trisomy 18 alongside a normal male fetus. In both situations, the parents opted for pregnancy termination due to medical reasons. We must remember that in the case of a vanishing twin, the blood collection for NIPT must be delayed by eight weeks after this discovery is made. 

### 3.5. NIPT-Identified High-Risk Cases

**Figure 4:**
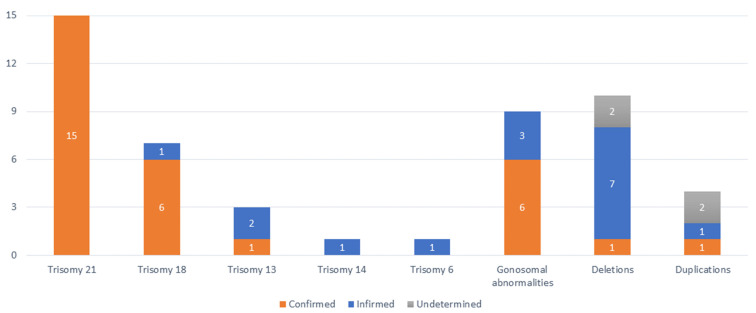
Distribution of cases according to the risk identified at NIPT (aneuploidies and CNVs), with confirmed and denied cases highlighted.

The types of anomalies identified, along with the number of confirmed, unconfirmed or unidentified cases are presented in Figure 4. The study found a 2.57% (36/1400) occurrence of high-risk for aneuploidy and observed an elevated risk for CNVs (microdeletion/duplication syndromes) in 1% (14/1400) of cases. The risk of combined anomalies, involving one aneuploidy and one microdeletion, was identified in 0.14% (2/1400) of cases. In two cases with a risk of microdeletion and two cases with a risk of microduplication, no verification tests were conducted based on parental decisions, leading to the continuation of pregnancies (Figure 4).

The only abnormality for which the NIPT risk result was fully confirmed, with a PPV of 100%, was Trisomy 21 (Down syndrome). For trisomy 18 (Edwards syndrome), confirmation occurred in 85.71% (6/7) of cases. Only 33.33% (1/3) of instances confirmed the risk for trisomy 13 (Patau syndrome) in the studied cases. The two cases with rare trisomy (+14 and +6) were false positives. Confirmation of gonosomal aneuploidies occurred in 66.66% (6/9) of cases (Table 1). The risk of monosomy X (Turner syndrome) was identified in four cases but confirmed in 50% of instances. Three cases of disomy of the Y chromosome (Jacobs syndrome) followed, all confirmed. The case of Klinefelter syndrome proved to be a variant of the syndrome with karyotype 48,XXXY. Trisomy X was a false positive.

Regarding microdeletions (Table 2), ten cases were reported, of which in two cases the pregnancies continued without further analysis, remaining undetermined at the request of the parents. In seven cases, the microarray method was performed, and these turned out to be false positives. Only one case with a deletion of 54.27 Mb of the Xp arm was confirmed. Duplication risk occurred in four cases: two remained undetermined, one proved false positive, and one was confirmed: a 7.5 Mb duplication of 9p. In three cases (two deletion and one duplication), we found that the CNVs were of maternal origin (Table 2).

### 3.6. Gestational Age at Time of NIPT 

In the NIPT analysis databases, the gestational age (GA) at the time of maternal blood sampling is written in whole weeks, and we proceeded similarly. In our study group, the average gestational age was 12.025 weeks (range: 10–23 weeks), with slight variations of 12.88 weeks (range: 10–23 weeks) for the high-risk group and 12.67 weeks (range: 10–22 weeks) for cases confirmed through an invasive method. Most cases were observed shortly after reaching the gestational age of 10 weeks (Table 3).

### 3.7. Fetal Fraction 

The percentage (%) of cffDNA in maternal plasma is the fetal fraction (FF). NIPT requires a minimum FF of 3.5% for an interpretable result. In our study, FF in the entire study group was, on average, 10.65% (range: 3.5–28.12%), while in NIPT high-risk pregnancies, FF was higher, on average 12.88% (range: 3.5–41.94%) (Table 3).

### 3.8. Maternal Age

The mean maternal age in the entire group was 33.1 years (range: 17–47). Patients with an increased risk at NIPT had an average maternal age of 35.35 years, while for those with confirmed risk, the average maternal age was 35.82 ( Table 3 and Table 4).

### 3.9. Indications for the NIPT

Maternal age prompted genetic testing, and 17 out of 28 cases (60.71%) were confirmed, while 10 were false positives. In our study group, more than half of the cases were conducted immediately after the 10th week of pregnancy to check the apparently normal pregnancy. Of these, 14 had an increased risk at NIPT, with a confirmation rate of 57.14% (8/14). The biochemical risk assessment revealed eight instances of increased risk and 75% (6/8) were confirmed (Table A1). Biochemical risk was assessed in seven cases in the NIPT risk group. We observed that in 42.85% (3/7) cases the double test (DT) and NIPT risk was confirmed, in 14.28% (1/7) cases the trisomy risk was unconfirmed in both DT and NIPT tests, 14.28% (1/7) cases remained undetermined, and in 28.57% (2/7) cases the NIPT risk was confirmed and the biochemical risk was unconfirmed. A superiority of the NIPT test to assess risk compared to biochemical risk is visible.

In the case of abnormal ultrasounds, it should be mentioned that all cases with high risk at NIPT were confirmed. There were several cases in which the ultrasonography identified congenital malformations, the NIPT test did not find a high risk, but the congenital anomalies were present and had another etiology. In some cases, there were several risks that were clinically stable before performing the NIPT analysis. The main indications of the NIPT test in our study are presented in Table 4.

### 3.10. The Direct Method of Checking High-Risk Pregnancies at NIPT

**Figure 5:**
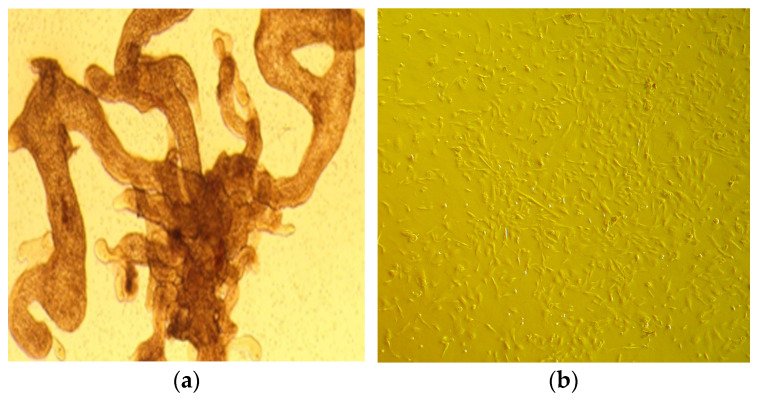
Inverted microscope images were used to observe the long-term growth of the cells: (**a**) chorial villi obtained from one of the arrested pregnancies; (**b**) amniotic cells obtained by amniocentesis placed in long-term culture under temperature-controlled conditions with 5% CO_2_ and photographed after 12 days.

**Figure 6:**
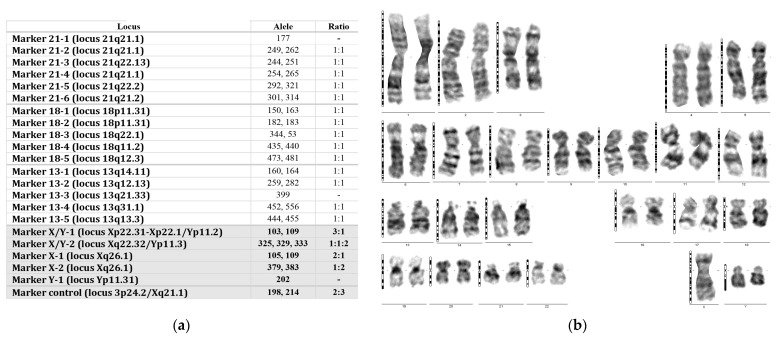
Aneuploidies of sex chromosomes confirmed: (**a**) QF-PCR result obtained from cells obtained via chorionic villus sampling revealing trisomy X in a male fetus (the shading indicates the abnormal part of the result), (**b**) fetal karyotype with approximately 450 bands/haploid set obtained from amniotic cells revealing disomy Y (Jacobs syndrome). The shading indicates the abnormal part of the result.

The results showed 47 cases with high risk. For four of the CNVs risk cases, there were no further investigations. Four pregnancies miscarried before NIPT results were available and further genetic testing was only possible using aborted placental material. Medical professionals conducted a total of 11 CVS and 26 amniocentesis procedures to collect biological material directly from the fetus for the necessary additional genetic testing for high-risk pregnancies. Long-term culture of amniocytes or cells from chorionic villi was necessary to acquire the fetal karyotype (Figure 5, Figure 6b and Figure 8).

**Figure 7:**
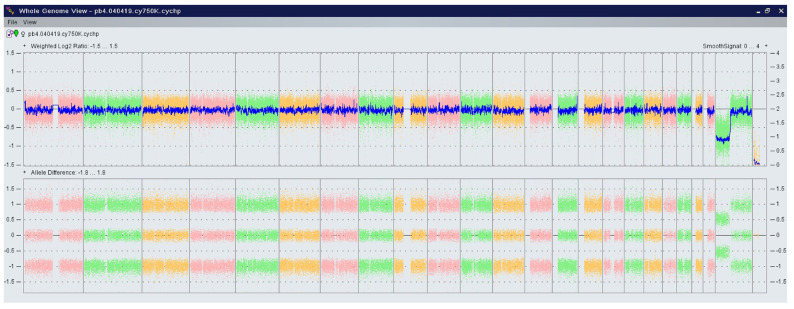
Chromosome copy number profiles for the sample which was a female fetus with a large deletion on Xp chromosome (microarray analysis).

**Figure 8:**
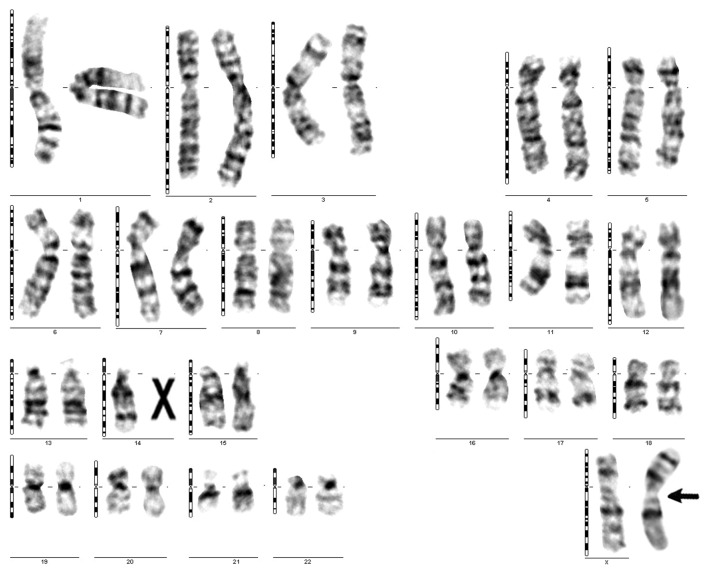
Karyotype obtained from amniotic cells performed as a confirmation method for combined NIPT risk of trisomy 14 and Xp chromosome deletion. The arrow indicates the existence of a derivative chromosome obtained by the translocation of chromosome 14 on the Xq chromosome at the level of the centromere. The karyotype confirms the existence of the Xp deletion but no trisomy 14 was found.

We performed the QF-PCR method and/or fetal karyotypes to identify aneuploidies (Figure 6). Microarray analysis has shed light on the risk of CNVs. Only two cases were confirmed: Dup9(p24.3-p21.1) and Del(X)(p22.33-11.21). In this case with Del(Xp), we also examined the fetal karyotype, which indicated the existence of a translocation between chromosome 14 and the long arm of the X chromosome, with deletion of the entire Xp arm. De novo Fetal microarray analysis (Figure 7) and fetal karyotype (Figure 8) illustrate this atypical case. This case had a combined risk at NIPT: confirmed Xp deletion and trisomy 14 false positive.

## 4. Discussions

The conventional approach for pregnancies, consisting of an ultrasound examination and serum markers (double test), is performed during weeks 11–13 of pregnancy [11]. Conventional combined screening is the official approach in Romania. Since 2017, Belgium and The Netherlands have successfully implemented NIPT as a first-line test for all pregnant women before a late first trimester ultrasound [12]. Starting in 2019, Germany, France, and England have introduced publicly funded NIPT as a second-line test for common chromosomal aneuploidies (trisomy 21, 18, and 13) [13]. Reproductive autonomy is valued across countries but interpreted and implemented differently: England emphasizes informed consent and personal choice; France places importance on medical information and state protection; while Germany focuses on balancing the ‘right to know’ and the ‘right not to know’ [13]. Despite differences in the risk cut-offs for considering pregnancy as high risk (e.g., in France, the cut-off risk is ≥1:51, while in England, it is ≥1:150), the primary discussion concentrates around the criteria for reimbursing the test. In Russia, a pilot study in 2022 in Moscow considered a cut-off risk of ≥1:100, with the intention of deciding whether to use NIPT as second-line screening in the first trimester [14]. In Romania, despite patients covering the entire cost of the genetic testing, we observe an increase in the number of tests (Figure 2) and an increased interest from pregnant women. For the Nifty test (BGI), special insurance allows patients with a high-risk result at NIPT to be fully reimbursed for all genetic tests and invasive procedures. Considering that in Romania non-invasive genetic tests and the microarray method are not covered by the national insurance system, this possibility to check for pregnancies at risk using NIPT is a great financial help.

The ongoing study is the second one conducted in Romania, focusing on a case load of 1400 cases. It follows the previous survey [3], which presented the genetic counseling and management of 380 cases. The current study compares NIPT high-risk cases with confirmed positive cases. This approach enables us to identify false positive and false negative instances, improving our ability to make more accurate predictions in the future and to address the numerous questions raised by couples. Romania’s national health insurance system does not cover NIPT tests, but individuals can obtain them commercially. An increasing number of couples opt for these tests directly due to their non-invasiveness and high accuracy compared to biochemical screening tests. If significant abnormalities or known minor fetal markers are detected via ultrasound, it is advisable to proceed directly to invasive tests, as our study shows a 100% probability of confirming the abnormalities found via NIPT, similar to other studies that have reported a predictive value 98.9% positive [15].

We consider that NIPT testing could be the first choice for all pregnancies regardless of maternal age or other indications. Advanced maternal age (over 35 years) remains a valuable indicator for assessing the risk of a pregnancy, especially for aneuploidy. However, its use as the sole indication for NIPT screening has a very low sensitivity (30%) and an important false positive rate (15%) [16]. In our study, 46% (17/37) of cases confirmed positive for aneuploidy had a maternal age over 35 years ( Table 3 and Table 4). Conversely, in cases with high risk at NIPT and advanced maternal age (>35 years), there were 60.7% confirmed cases and 39.3% false positives. Compared to normal pregnant women who came for pregnancy check-ups, there were 57.14% confirmed positive and 42.86% false positive cases. Although maternal age has been an essential indicator for prenatal screening for a long time [17], the current study shows that NIPT testing is a scientifically reliable choice for any pregnancy.

To date, there are retrospective as well as prospective studies and meta-analyses, each totaling around 150 publications, on NIPT demonstrating the high efficacy of cffDNA in prenatal screening for fetal trisomy 21, 18, 13, and monosomy X [18]. A recent review published in 2023 shows that the global implementation of NIPT led to the implementation of these tests in national healthcare programs on all continents [19].

Studies on NIPT have been published on a vast number of cases. In China, 14,574 pregnant women have been tested to assess the accuracy of NIPT results for twins, concluding that efficiency is similar to that of singleton pregnancies [20,21]. We had 11/13 cases of twin pregnancy with normal results for common aneuploidies. Two cases had abnormal results with a risk of trisomy 18 and trisomy 21, which were confirmed in one fetus in each case.

Knowing from previous studies [14] that a low FF can lead to inconclusive results in older women (>35 years), significantly higher weight and increased BMI, we recommended delaying the test from week 10 to week 12 and we obtained conclusive results in almost all cases. Only 0.14% (2/1400) cases remained without NIPT results after a new collection. 

While most tested pregnancies occurred spontaneously, we also documented 85 pregnancies that resulted from medically assisted reproduction. These couples are particularly keen to test via non-invasive methods pregnancies obtained with difficulty.

All 15 cases at risk for trisomy 21 were confirmed, two of them stopping in evolution (Table 1). This is in accordance with other studies, the PPV for trisomy 21 being almost 100% in all studies [22,23]. The majority (6/7 cases) of trisomy 18 cases were also confirmed. The false positive case of trisomy 18 involved a 44-year-old mother with an FF of 5% at a pregnancy age of 15 weeks and resulted in a continued pregnancy with the birth of a normal baby. Regarding trisomy 13, three cases were identified; one was confirmed, and two were negative (ages 33 and 39 with biochemical risk for trisomy 21, FF 12% and 14%, respectively). We observed negative correlations, just as for the two cases with rare trisomy (trisomy 6, trisomy 14) that proved to be false positives. We have not dismissed the possibility that these abnormalities might be identifiable from the earliest stages of pregnancy, as there has been a reported case of high-level trisomy 14 mosaic detected during amniocentesis in a pregnancy with congenital heart defects and intrauterine growth restriction observed during fetal ultrasound [24].

The results regarding sex chromosome aneuploidies were surprising. Although the prevalence rate is the lowest among these types of aneuploidies, namely 1 in 3000 [25], we had three cases of Y disomy, all confirmed. One case of Klinefelter syndrome (XXY) turned out to be a rarer variant of type XXXY syndrome, and the family decided to terminate the pregnancy. The only case of trisomy X was false positive. There were four cases of monosomy X of which 50% were confirmed; in these two cases the pregnancy was stopped in evolution and the analysis was performed from the aborted product.

Unexpectedly, four pregnancies stopped progressing before the NIPT result was available, 50% of them having a risk for trisomy 21 (two cases) and 50% a risk for monosomy X (two cases). In arrested pregnancies, chromosomal abnormalities of the products of conception are present in more than 50% of cases [26]. For pregnant women, it is usually difficult to decide to terminate the pregnancy even if they have a confirmed positive result; but knowing the cause of fetal death for the management of future pregnancies was appreciated. Another unexpected result was the existence of the late diagnosis of triploidy, around week 18, in two pregnancies that had normal NIPT results, so they were considered false negative cases. Triploidy, although considered a rare abnormality, has a high frequency in arrested pregnancies [27,28].

We chose these NIPT tests for the broad possibility of testing for CNVs, primarily to additionally exclude this risk, which is small but still not negligible, especially as we have been part of a research collective with the purpose of diagnosis and publication of several articles with case presentations and reviews of people affected by these conditions [28,29,30,31].

Conditions such as microdeletions-duplications are rarely diagnosed, but their prevalence has not been clearly defined [5]. In our study group, there were ten cases at risk for microdeletions (Table 2), of which nine were unconfirmed and only one was confirmed positive. Two cases of DiGeorge syndrome, two cases of Prader Willi syndrome, one case of Lejeune syndrome, and one case of Del 1p36 were unconfirmed by microarray. 

Cases of 20q deletion have also been reported. A case with a 7.5 Mb 20q13.2-q13.33 deletion was reported that presented pre- and post-natal growth retardation, with absent speech, intellectual disability, hypotonia, and a unilateral cleft lip [32]. Another reported case had a de novo 20q11.2 microdeletion, measuring 1.2 Mb, which resulted in intellectual disability and dysmorphic features [33]. In our group we had a special situation, more precisely two consecutive pregnancies of the same woman, where the 20q deletion was identified, with diameters of 18.4 M and 19.5 M. In both pregnancies, microarray excluded the deletions, and two normal children were born. Upon re-evaluation at BGI headquarters, we discovered that the pregnant woman who was physically and mentally normal, had mosaicism, with a 7% clone with 20q deletion. Deletions on the long arm of chromosome 20, del(20q), are common karyotypic abnormalities in myeloid disorders but subclonal mosaic events of a region implicated in myeloid disorders on 20q are events that can be tolerated until additional events drive myeloid disorders accumulate [34].

The case with risk of Del (10q25.2–q26.3), although it had a size of 22.57 Mb, proved to be false positive. There have also been reports of prenatal diagnosed cases with interstitial deletion of del(10)(q25.2q25.3~26.11). In one case, the deletion comprises 6-Mb and is associated with hemizygosity for 30 genes. Postmortem physical examination and autopsy of the fetus, after medical termination of pregnancy at 20 weeks, revealed pronounced microretrogenism and hypertelorism, crooked legs, as well as minor internal abnormalities such as annular pancreas, atypical lobed liver and absence of cholecystitis [35].

Also, another case with Dup 22q11.21 was proven to be of maternal origin. Hence, CNVs are a crucial source of regular and pathogenic genome variations.

A previous study evaluated all cytogenetic-phenotypic aspects in a cohort of 47 Japanese women with phenotypic changes such as short stature, short neck, cubitus valgus, lymphedema, and gonadal dysfunction [36]. In our case, the pregnant woman was 41 years old; she had previously had a fetus with Down syndrome, for which she stopped the pregnancy. In the current pregnancy, she performed both the fetal karyotype, which indicated the existence of a translocation between chromosome 14 (14q) and the long arm of the X chromosome, and the microarray, which documented the 54.27 Mb deletion of the short arm of the X (Xp) chromosome ( Figure 7 and Figure 8). De novo translocations can cause deletions in the offspring, causing well-known syndromes with related risks [37]; in our case, a variant of Turner syndrome resulted.

In our group, we had four cases of microduplications. The case with Dup 5q23 risk remained undetermined and the pregnancy continued. The case with Dup9(p24.3-p21.1) was confirmed by the microarray analysis and because the ultrasound showed an important fetal growth restriction observable starting with the 18th week of pregnancy, the parents decided to terminate the pregnancy. Two cases with Dup 22q11.21 in the region specific to DiGeorge syndrome were also identified at NIPT. One case was described as atypical and proved to be of maternal origin, a false positive for the fetus. The second case remained undetermined. Both pregnancies continued and children were born without dysmorphic features at birth. Individuals experiencing a duplication in the 22q11.21 region might exhibit intellectual or learning challenges, developmental delays, sluggish growth resulting in short stature, and hypotonia. However, it is noteworthy that many individuals with this condition do not manifest any evident physical or intellectual disabilities [38]. Genetic counseling is difficult in these situations, where few cases are reported, often with varying results [30,31,39].

A 2021 study of blastocysts evaluated by non-invasive preimplantation genetic testing (PGT), identified an extremely high number of embryos with CNVs abnormalities on almost all chromosomes. In order of frequency the following abnormalities were identified: dup(4q), del(5q), del(9q), dup(6q), del(2p) [40]. This wide variability of abnormalities proven to be present in IVF embryos is an additional reason for choosing an extensive NIPT test to be presented to patients as a possible alternative to a more restrained test targeting only common trisomies.

NIPT has come with new challenges, especially economic issues and ethical implications [5]. Advantages of NIPT tests:

- They are performed early in pregnancy (from the 10th week onwards).
- They are more accurate in detecting common chromosomal aneuploidy, with a lower rate of false positives, by comparison with biochemical screening tests.
- They reduce the number of invasive tests and consequent risk of miscarriage.
- We would like to mention the disadvantages of the NIPT compared to the invasive techniques:
- NIPT has not yet become a diagnostic method and women may still need to undergo risky invasive procedures to verify a possible positive NIPT finding [41].
- There is concern that the use of NIPT in routine prenatal care may increase the risk of stigma and discrimination against individuals who are living with trisomy 21, negatively impacting the support society provides to women who decide to raise a child with Down syndrome.
- There is a risk of expanding screening for “less severe” conditions and prenatal sex selection [42,43].

Regardless of how one approaches an NIPT test, obtaining informed consent [44] is necessary after a genetic consultation conducted by a health professional, preferably a geneticist. In our case, every pregnant woman received a pre-test genetic consultation and a second consultation after receiving the test result. We conducted a third genetic counseling session for cases requiring an invasive method to check for an NIPT-identified risk. The extensive initiative, which involves providing sufficient information, explaining diverse options, and offering support, is designed to help women make informed decisions, and their choices should be honored.

Ethically, each country balances reproductive autonomy with other values, such as the rights of people with disabilities, human dignity, and the health professional’s duty to care for each patient [13]. The term “routinization” of prenatal diagnosis was introduced, linking it to informed choice and a eugenic attitude. From a psychological and sociological perspective, one must assess all aspects, considering it a heterogeneous phenomenon with widely varying approaches depending on the country in which it is discussed [45].

The value of this study lies in highlighting the particular problems, the atypical cases identified during prenatal screening and diagnosis, and how the multidisciplinary effort of the authors elucidated them. The expansion of CNV assessment panels uncovers many conditions and diseases that are still too little known [5]. Therefore, each presentation of rare cases generates new information, enabling researchers in this field to build a more solid foundation of scientific knowledge. Globalization has allowed NIPT tests to be carried out on millions of pregnant women worldwide, so disseminating scientific information at the same level allows us to access the world’s scientific knowledge. The ethical implications remain a challenge that depends on the socio-cultural context of each country and here numerous approaches are allowed.

## 5. Conclusions

This study allowed us to explore the accessibility of and interest in NIPT testing among pregnant women in western Romania, to follow up on managing high-risk cases, and to draw conclusions for 1400 cases. The presentation of cases considered rare or atypical is valuable for researchers involved in this field. Invasive diagnostic procedures have been considered and remain the “gold standard” in prenatal diagnosis. A combination of NIPT, prenatal ultrasonography, fetal karyotype, microarray, and genetic counseling is helpful for prenatal screening and diagnosis. We advocate for early testing of pregnancies using a non-invasive method. In instances where a potential risk is identified but not confirmed (i.e., a false positive result), our results show that pregnant women in Romania express happiness that they can proceed with the pregnancy. They harbor no resentment due to being tested through an invasive procedure. The clinical advantages of NIPT tests are undeniably valuable, and it would be beneficial to expand their application to as many pregnancies as possible, irrespective of reimbursement considerations. Given the socio-cultural context of our country, we believe that all pregnancies should be recommended for NIPT screening, accompanied by expert pre- and post-test genetic counseling.

## Figures and Tables

**Table 1:** The risk of aneuploidy at NIPT.

Chromosomal Abnormality	Type of Aneuploidy	Suspected Risk at NIPT^1^	Confirmed Risk at NIPT
Common autosomal trisomies	Trisomy 21	15	15
	Trisomy 18	7	6
	Trisomy 13	3	1
Rare autosomal trisomies	Trisomy 14	1	0
	Trisomy 6	1	0
Gonosomal aneuploidy:	Y chromosome disomy	3	3
	Klinefelter XXY	1	1 (XXXY)
	Trisomy XXX	1	0
	Monosomy X	4	2

NIPT^1^ = Non-Invasive Prenatal Testing.

**Table 2:** The risk of CNVs at NIPT.

CNVs	Size	Status	Origin
Del^1^1p36	2.30 Mb^3^	False positive	
Del5p (Lejeune)	Undetermined	False positive	
Del(10q25.2–q26.3)	22.57 Mb	False positive	
Del(15)(q11.2–13.1) (Prader Willi)	Undetermined	Undetermined	
Del(15)(q11.2–13.1) (Prader Willi)	5.60 Mb	False positive	
Del(20q11.21–q13.13)	18.40 Mb	False positive	MATERNAL
Del(20q11.21–q13.13)	19.50 Mb	False positive	MATERNAL
Del(22q11.21) (DiGeorge)	2.54 Mb	False positive	
Del(22q11.21) (DiGeorge)	Undetermined	Undetermined	
**Del(X)(p22.33–11.21)**	**54.27 Mb**	**Positive: arr [GRCh37] Xp22.33p11.1 (168551_62051248) x 1**	**FETAL**
Dup(5q23)	?	Undetermined	?
Dup^2^(22q11.21) (DiGeorge)	?	Undetermined	?
Dup(22q11.21) (DiGeorge)	atipical	False positive	MATERNAL
**Dup(9)(p24.3-p21.1)**	**7.5 Mb**	**Positive: arr [GRCh37]** **9p24.3p21.1 (208455_28238046) x 3**	**FETAL**

Del^1^ = Deletion, Dup^2^ = Duplication, Mb^3^ = Megabase. ? = Unknown.

**Table 3:** Comparative presentation of gestational age, fetal fraction, and maternal age in our group.

Evaluated Parameter	Study Group	Suspected Risk by NIPT	Confirmed Risk by Direct Method
Average GA^1^ (weeks):	NIPT^4^: 12.025	NIPT: 12.88	NIPT: 12.67
	Nifty^6^-pro: 12.04	Nifty-pro:12.65	Nifty-pro:12.41
	NIPS^5^: 12.01	NIPS: 13.12	NIPS: 12.93
Average FF^2^ (%)	NIPT: 10.65	NIPT: 12.88	NIPT: 11.67
	Nifty-pro: 10.98	Nifty-pro: 12.46	Nifty-pro: 11.22
	NIPS: 10.30	NIPS: 12.12	NIPS: 12.12
Average MA^3^ (years):	NIPT: 33.1	NIPT: 35.35	NIPT: 35.82
	Nifty-pro: 33.5	Nifty-pro: 35.7	Nifty-pro: 36
	NIPS: 32.7	NIPS: 35	NIPS: 35.65

GA^1^ = Gestational Age, FF^2^ = Fetal Fraction, MA^3^ = Maternal Age, NIPT^4^ = Non-Invasive Prenatal Testing, NIPS^5^ = Non-Invasive Prenatal Screening, Nifty^6^ = Non-Invasive Fetal Trisom Y.

**Table 4:** The main indications identified in NIPT risk cases.

	Cases with High-Risk at NIPT^1^	Cases with Confirmed Risk	Unconfirmed Cases (False Positives)	Indefinite
Maternal age	28	17 (60.71%)	10	1
Monitoring the apparently normal pregnancy	14	8 (57.14%)	6	0
Biochemical risk	8	6 (75%)	1	1
Abnormal ultrasound	5	5 (100%)	0	0

NIPT^1^ = Non-Invasive Prenatal Testing.

## Data Availability

The data used is not currently in any national or international database. The data comes from a private genetics clinic in Romania (Medical Genetics Office Dr. Gug), and are currently not available to the general public.

## Abreviations

ABI GeneAmp^®^ System 9700 (Thermo Fisher Scientific Inc. (NYSE: TMO) Waltham, Massachusetts, United States)	Applied Biosystem Gene Amplification
ACOG	American College of Obstetricians and Gynecologists
BCT	Blood Collection Tube
BGI	Beijing Genomics Institute
cffDNA	Cell-Free Fetal Deoxyribonucleic Acid
CNV	Copy number Variation
CVS	Chorionic Villus Sampling
DECIPHER	DatabasE of Chromosomal Imbalance and Phenotype in Humans using Ensembl Resources
CNV	Copy number Variation
CVS	Chorionic Villus Sampling
DECIPHER	DatabasE of Chromosomal Imbalance and Phenotype in Humans using Ensembl Resources
Del	Deletion
DNA	Deoxyribonucleic Acid
DT	Double Test
Dup	Duplication
FF	Fetal Fraction
GA	Gestational Age
IVF	In Vitro Fertilization
MA	Maternal Age
Mb	Megabase
NCBI	National Center for Biotechnology Information
NGS	Next-Generation Sequencing
NIPT	Non-Invasive Prenatal Testing
NIFTY	Non-Invasive Fetal TrisomY
NPV	Negative Predicitive Value
OMIM	Online Mendelian Inheritance in Man
QF-PCR	Quantitative Fluorescent Polymerase Chain Reaction
PPV	Positive Predictive Value
SHOX gene	Short-Stature Homeobox gene
SNP-Array	Single Nucleotide Polymorphism Array
TT	Triple Test
USA	United States of America

## Appendix A

**Table A1:** Biochemical risk, represented by Double Test (DT) and Triple Test, correlated with other clinical data.

**No**	**Maternal Age**	**Gestational Age**	**Biochemical Risk**	**FF**	**NIPT Risk**	**Invasive Procedure**	**Genetic Test**	**Conclusion**	**Pregnancy Management**
1	31	19	TT^1^ = 1:100 T21^2^ risk	15%	NIPS^4^ risk: XYY	Amniocentesis	QF-PCR^8^: XYY	NIPT Confirmed TT infirmed	Continues
2	28	11	DT^3^ = 1:5 T21 risk	25%	NIPS risk: T21	Uterine evacuation	Kariotype: T21	NIPT Confirmed DT Confirmed	Miscarriage
3	33	12	DT = 1:300 T21 risk	14%	NIPS risk: T13	Amniocentesis	QF-PCR^8^: **XY**	NIPT Infirmed DT Infirmed	Continues
4	29	16	DT = 1:140 T21 risk	22%	NIPS risk: Del^5^622q11	Amniocentesis	Array: XY	Undetermined	Continues
5	41	13	DT = 1:4 T21 risk	15.6%	NIFTY^6^ doble risk: T14^7^ + DelXp	Amniocentesis	Kariotype + Array	NIPT Confirmed (delXp) DT Infirmed	Medically terminated pregnancy
6	25	12	DT = 1:141 T21 risk	12%	Nifty risk: T21 risk	Amniocentesis	Kariotype	NIPT Confirmed DT Confirmed	Medically terminated pregnancy
7	36	12	DT = 1:70 T21 risk	13%	Nifty risk: T21 (47,XY + 21) +(46,XX)	Double amniocentesis	QF-PCR + Kariotype	NIPT Confirmed DT Confirmed	Medically terminated pregnancy

TT^1^ = Triple Test, T21^2^ = Trisomy 21, DT^3^ = Double Test, NIPS^4^ = Non-Invasive Prenatal Screening, Del^5^ = Deletion, Nifty^6^ = Non-Invasive Fetal TrisomY, T14^7^ = Trisomy 14, QF-PCR^8^ = Quantitative Fluorescent Polymerase Chain Reaction.
